# Occupational Health Barriers in South Africa: A Call for Ubuntu

**DOI:** 10.5334/aogh.4424

**Published:** 2024-05-28

**Authors:** Muzimkhulu Zungu, Jerry Spiegel, Annalee Yassi, Dingani Moyo, Kuku Voyi

**Affiliations:** 1 National Institute for Occupational Health, a division of the National Health Laboratory Service, Johannesburg 2000, ZA; 2 School of Health Systems and Public Health, Faculty of Health Sciences, University of Pretoria, Pretoria, South Africa; 3 Department of Public Health, Faculty of Health Sciences, Walter Sisulu University, Mthatha, South Africa; 4 School of Population and Public Health, University of British Columbia, Vancouver, British Columbia, V6T 1Z3, Canada; 5 School of Public Health, University of the Witwatersrand, Johannesburg, South Africa; 6 Faculty of Community Medicine, National University of Science and Technology, Bulawayo, Zimbabwe; 7 Baines Occupational Health Services, Harare, Zimbabwe

**Keywords:** Occupational health services, barriers, health workers, Ubuntu

## Abstract

Many low- and middle-income countries (LMICs) grapple with shortages of health workers, a crucial component of robust health systems. The COVID-19 pandemic underscored the imperative for appropriate staffing of health systems and the occupational health (OH) threats to health workers. Issues related to accessibility, coverage, and utilization of OH services in public sector health facilities within LMICs were particularly accentuated during the pandemic. This paper draws on the observations and experiences of researchers engaged in an international collaboration to consider how the South African concept of Ubuntu provides a promising way to understand and address the challenges encountered in establishing and sustaining OH services in public sector health facilities. Throughout the COVID-19 pandemic, the collaborators actively participated in implementing and studying OH and infection prevention and control measures for health workers in South Africa and internationally as part of the World Health Organizations’ Collaborating Centres for Occupational Health. The study identified obstacles in establishing, providing, maintaining and sustaining such measures during the pandemic. These challenges were attributed to lack of leadership/stewardship, inadequate use of intelligence systems for decision-making, ineffective health and safety committees, inactive trade unions, and the strain on occupational health professionals who were incapacitated and overworked. These shortcomings are, in part, linked to the absence of the Ubuntu philosophy in implementation and sustenance of OH services in LMICs.

## Introduction

Health workers (HWs), are defined by the World Health Organization (WHO) as all people engaged in work actions whose primary intent is to improve health, including clinicians and non-clinicians such as managers and support workers [1]. The WHO lists the health workforce as one of the six building blocks of a health system and is an essential component for a resilient health system [2]. However, health systems globally continue to face HW shortages, which are at a crisis level for many low- and middle-income countries (LMICs), aggravating health inequities [3]. More concerning are reports that while there are HW shortages, HWs themselves neglect their own health and well-being in the midst of increased workloads, stress and burnout [4].

Workers in LMICs are reported to experience the least availability and inclusiveness with regards to occupational health (OH) services [5]. Such services primarily focus on preventive interventions (see Box 1) as well as providing advice to the employer, the workers and their representatives on the requirements for establishing and maintaining a safe and healthy working environment [6]. In South Africa, Moodley et al. reported that only 60% of HWs who work in public sector health facilities had access to occupational health clinics [7]. Availability of OH clinics for HWs, however, does not always mean HW access, coverage and utilization of effective OH interventions. Moreover, it is important to ensure that OH interventions for HWs are effective and accessible, as OH is a human right [8] and its provision aligns well with how the philosophy of Ubuntu can be applied in the workplace setting [9]. Ubuntu an African orientation that has been receiving increased global attention [10], according to Msila [11] is sacrificing for others selflessly, caring and protecting fellow human beings.

**Box 1 Some interventions of an OHS**

*Adapted from the ILO C161 [6].

- Hazard identification and risk assessment;
- Advice on planning and organisation of work;
- Providing information, training, and education on OH;
- Surveillance of workers’ health in relation to work;
- Promoting the adaptation of work to the worker;
- Contribution to measures of vocational rehabilitation;
- Organising of first aid and emergency treatment;
- Reporting and analysis of occupational accidents and diseases;
- Establishment of Health and Safety Committees.

The coronavirus disease 2019 (COVID-19) pandemic highlighted the need for OH as well as infection prevention and control (IPC) measures for HWs not only in South Africa, but across the world [12]. Since the beginning of the COVID-19 pandemic, the authors have participated in studying and implementing interventions aimed at strengthening OH and IPC for HWs in South Africa and globally as part of an international collaboration, formed within the WHO Collaborating Centres for OH [13]. Our observations throughout the COVID-19 pandemic indicate that OH services for HWs are not given adequate priority, potentially violating the principles of Ubuntu philosophy. Here we provide this viewpoint to share our lived experiences about the challenges in establishing or implementing, maintaining and sustaining OH, and IPC measures for HWs in public healthcare facilities in South Africa during the COVID-19 pandemic [12,13,14,15].

## Projects Leading to This Viewpoint

While the authors have vast experience in establishing and implementing OH and IPC measures for HWs, the main source for the current viewpoint was during the period 2020–2023 wherein the authors, using participatory action research principles to strengthen OH, implemented a COVID-19 OH intervention study. This was a quasi-experimental study utilising a mixed methodological approach, which compared changes in OH systems using the WHO’s six building blocks of a health systems [16], pre versus post with two concurrent interventions, namely the occupational health and safety information system (OHASIS) [15] and International Labour Organization (ILO) and WHO’s HealthWISE tool [14].

## Observation of an OH Culture That Lacks Ubuntu

The implementation and uptake of OH services offered to any population group, and in our case, public sector HWs, is dependent on the population’s OH culture. Occupational health culture is the set of shared attitudes, values, beliefs, and behaviours [17]. For HWs in South Africa’s public sector health facilities, our lived experiences suggest that the prevailing culture is primarily orientated to practice ‘ticking the box’ when it comes to OH. By this we mean participating in OH administrative activities so that when audited it would seem like a facility has functional and effective OH services. We argue that this type of OH culture lacks Ubuntu. Applied to OH services, Ubuntu means OH stakeholders (government, employers, trade unions, and workers themselves) caring enough to proactively promote and protect the health and safety of workers. Hence, the challenges we observed and discussed here, which in part led to delayed and sometimes failed establishment or implementation of OH interventions, such as OHASIS interventional research conducted by the authors, relate to the deviation from Ubuntu within OH.

### Challenges Facing a Positive OH Culture

Our focus is on challenges at a facility level, with emphasis on what contributes to the failures to provide effective access, coverage and utilization of OH services to positively impact the health and well-being of HWs.

- Stewardship, arguably the most complex but critical aspect [16], is an ethical value that embodies the strategic guidance and oversight for planning and management of OH resources [18]. This, we observed, is the most important influence on whether HWs have access, coverage, and utilize functional and effective OH services. Top management aligned with trade unions, is responsible for stewardship, which requires political and technical acumen and actions. Top management should seek the participation of provincial political and administrative leadership in OH, as this would increase the financial and human resources for OH as well as be visible and participate actively in OH, so as to lead by example. These leadership and stewardship roles have proven to be lacking based on our observation and where present, OH resources, access, coverage and utilization were notable higher.
  Further, we observed that passive leadership and stewardship on OH services by top management and trade union representatives negatively affect the implementation of OH interventions. Since management is accountable for OH, the continued lack of use of effective intelligence systems for OH decision-making is another challenge [15], which is compounded by the lack of appropriate procedures in OH governance systems.
  Although South Africa is often said to have ‘good laws/and or policies for OH’ [14,19], these are mostly driven by a top–down approach. During the COVID-19 pandemic, facilities in our research projects received COVID-19 policies and plans prepared by provincial departments of health [14] that were then distributed within each health facility by the OH or IPC coordinators to unit or ward managers along with other documents. These were not always reviewed at the local facility level to ascertain their relevance and to provide education and training for HWs; in a nutshell, the culture of ‘ticking the box’ was fulfilled and the facility leaders and/or stewards did not oversee, monitor or evaluate the implementation of the policies and procedures outlined in the documents.
- Access to OH services is a right recognized by the United Nations, and it is the responsibility of the employer to provide and fund [8,20]. However, in our setting, there are no clearly ring-fenced budgets for OH, and managers do not budget for OH services [14,15]. Furthermore, while facilities may have OH professionals rendering services, they are not always present in sufficient numbers or adequately trained. In our setting, even wealthier facilities, OH staffing was often much lower than international norms [21] and nurse-driven. These OH nurses were competent, passionate, and hardworking, but were often overwhelmed and overworked, including having responsibilities for other programmes.
- In South African law, each workplace has to nominate, elect, and appoint health and safety representatives, and where at least two representatives are in place, establish a health and safety committee [22]. The health and safety representatives and committees working with management are to initiate, develop, promote, and review OH measures; where present and effective these committees are associated with fewer health and safety incidents and citations [14]. At the height of the COVID-19 pandemic, we were part of a nationwide drive for the establishment, monitoring, and evaluating the functioning of health facilities’ health and safety committees [14,23]. While the committees were present, we found them to epitomize the culture of ‘ticking the box’, lacking bipartism (i.e., employer–worker collaboration), with the employer mostly not represented, and the committee members lacking training on their OH duties, even though training has been shown to be beneficial [24]. As an example, a general observation found that hazard identification and risk assessment reports were not conducted in consultation with health and safety representatives and trade unions, not presented at the committee and most committee members had never seen these reports, so were not in a position to implement the recommendations. Further, the trade unions tended to be silent even when present in these committees, suggesting that they may require empowerment in their role.
- Information and information systems on OH are generally poor in the facilities and unable to provide OH intelligence for decision-making [15]. An intervention study examining the installation, establishment, and utilization of an OH and safety information system failed to be implemented during the stipulated study period, further raising the question about the culture of OH [15].

Without rigorous research on the reasons for delayed uptake of the interventions, we can only offer our viewpoint regarding the lack of leadership and stewardship, lack of bipartism on OH, overworked and overwhelmed OH nurses, ineffective health and safety committees, and shortage of resources. We observed that HWs were not holding management accountable for OH, possibly because their trade unions were not active and providing communication to their members, and thus the HWs were not empowered to know their rights.

The Canadian members of our international team note that OH services for HWs are not necessarily strong in their facilities either. However, in the Canadian province where team members are based, there is indeed a functioning OH information system [25], and the much better-resourced public health and infection control infrastructure, combined with a much lower burden of disease, makes the deficiencies somewhat less consequential than is the case for LMICs. Indeed our WHO survey found that OH and IPC mitigating measures during the pandemic were much less acceptable in the African region compared to wealthy countries, especially those with less disparity [26]. As such, the urgency of addressing OH in LMICs needs to be seen as a high priority.

## Conclusion

Recognizing the importance of OH interventions in resource-limited settings with precarious working conditions for HWs, Ubuntu is essential for establishing, maintaining, and sustaining effective OH interventions. To achieve this, OH leadership and stewardship should be based on the use of effective intelligence systems for OH decision-making, functional health and safety committees with a strong bipartisan approach, with active trade union engagement as well as well-trained OH professionals.

## Data Accessibility Statements

All data related to the manuscript is available within the manuscript.
